# High serum C1q-binding adiponectin levels in male patients with acute coronary syndrome

**DOI:** 10.1186/1475-2840-13-9

**Published:** 2014-01-09

**Authors:** Ken Kishida, Yasuhiko Nakagawa, Hironori Kobayashi, Toru Mazaki, Hiroyoshi Yokoi, Koji Yanagi, Tohru Funahashi, Iichiro Shimomura

**Affiliations:** 1 Department of Metabolic Medicine, Graduate School of Medicine, Osaka University, Suita, Osaka, Japan; 2 Kishida Clinic, Toyonaka, Osaka, Japan; 3 Department of Research and Development, Diagnostic Division, Otsuka Pharmaceutical Co., Ltd., Tokushima, Tokushima, Japan; 4 Department of Cardiology, Kokura Memorial Hospital, Kokura, Fukuoka, Japan; 5 Department of Cardiology, Kenporen Osaka Central Hospital, Osaka, Osaka, Japan; 6 Department of Metabolism and Atherosclerlosis, Graduate School of Medicine, Osaka University, Suita, Osaka, Japan

**Keywords:** Adiponectin, C1q, C1q-binding adiponectin, Acute coronary syndrome

## Abstract

**Background:**

The complement system is part of the immune system in acute coronary syndrome (ACS). Adiponectin has anti-atherogenic and anti-inflammatory properties. Adiponectin and C1q form a protein complex in blood, and serum C1q binding adiponectin (C1q-APN) can be measured. We investigated the comparative evaluation of serum C1q-APN levels in males with ACS, stable angina pectoris (SAP) versus controls.

**Methods:**

The study subjects were 138 Japanese patients who underwent diagnostic coronary angiography. Blood total adiponectin (Total-APN), C1q-APN and C1q were measured by enzyme-linked immunosorbent assays. Patients were divided into three groups according to the clinical condition: ACS (n = 78), SAP (n = 41) or normal coronary (NC, n = 19) groups.

**Results:**

Serum C1q levels were significantly higher in the ACS group (54.9±1.2 μg/mL) than in the NC group (48.0±2.5 μg/mL). Although serum Total-APN levels were significantly lower in the SAP and ACS groups, compared with the NC group (7.0±0.5, 7.2±0.3, 10.6±2.0 μg/mL, respectively), serum C1q-APN levels were significantly higher in the ACS group than in the NC and SAP groups (112.1±4.1, 66.3±4.4, 65.7±2.9 units/mL, respectively).

**Conclusions:**

Patients with ACS had higher serum C1q-APN levels.

**Trial Registration:**

UMIN000002997

## Background

Disruption of atherosclerotic plaques with associated thrombus is responsible for the majority of the acute coronary syndrome (ACS). Plaque instability is related closely to the degree of inflammation. The complement system plays a part of role in acute phase ACS [1]. Adiponectin, an adipocyte-derived blood protein [2] is present abundantly in injured arteries [3,4] and has anti-inflammatory, anti- atherosclerotic properties [5,6] and anti-diabetic property [7,8]. Low levels of total-adiponectin (Total-APN) have been reported in patients with coronary artery disease (CAD), including angina and ACS [9-15]. Adiponectin circulates in blood in three major forms: trimer, hexamer, and high-molecular weight (HMW) forms [16]. However, the main form of blood adiponectin remains to be elucidated. We reported recently that adiponectin binds with C1q in human blood, and also developed a system to measure human serum C1q-binding adiponectin (C1q-APN) [17]. Serum C1q-APN/Total-APN ratio is a novel marker of the metabolic syndrome in male subjects [17]. Serum C1q-APN/Total-APN ratio correlated with polyvascular diseases and stable CAD in type 2 diabetics [18,19]. However, the characteristics of C1q-APN in ACS patients remain unclear. The aim of the present study was to clarify the comparative evaluation of serum C1q-APN levels in males with ACS, stable angina pectoris (SAP) versus controls.

## Methods

### Participants

The study (#UMIN000002997) subjects were consecutive 138 Japanese admitted-patients who underwent diagnostic coronary angiography for suspected CAD following chest pain and/or ischemic changes on the electrocardiogram, and also underwent measurement of fat distribution by computed tomography scan for measurement of adipose tissues that was classified to visceral and subcutaneous fat tissue compartment at Kokura Memorial Hospital, Kenporen Osaka Central Hospital and Department of Metabolic Medicine, Osaka University Hospital between April and September 2009. Patients who had major surgery or trauma, serious active infectious diseases, malignancies, and chronic inflammatory diseases including rheumatoid arthritis, osteoarthritis, and inflammatory bowel disease were excluded from the study. Patients treated with pioglitazone, which is known to increase serum levels of Total-APN [20] and C1q-APN [21], and those with renal dysfunction (creatinine >1.5 mg/dL) [22] were also excluded from the study. The Medical Ethics Committees of Osaka University, Kenporen Osaka Central Hospital and Kokura Memorial Hospital approved the study.

Patients were divided into three groups according to the clinical condition: ACS [acute myocardial infarction (AMI), unstable angina pectoris (UAP)], SAP and normal coronary group (NC). All coronary angiograms were evaluated by at least two experienced examiners. Patients of the ACS group comprised those with AMI or UAP, and included 68 patients with AMI, who underwent catheterization within 24 hours of the onset of chest pain. The diagnosis of AMI was based on clinical symptoms, ST segment elevation (STEMI) on the electrocardiogram with non-ST segment elevation acute myocardial infarction (NSTEMI), coronary angiographic findings (occlusion of main coronary artery branch with thrombolysis in myocardial infarction (TIMI) grade flow of 0, 1, or 2), and changes in serum creatine kinase concentrations (more than twofold increase from the upper limit of the normal range). STEMI was diagnosed when new or presumed new ST-segment elevation of at least 0.1 mV in two or more leads was observed in any lead or new left bundle branch block was found on the index or qualifying electrocardiogram with 1 positive cardiac biochemical marker of necrosis. NSTEMI was diagnosed in the presence of 1 positive cardiac biochemical marker of necrosis without new ST-segment elevation seen on the index or qualifying electrocardiogram. UAP was diagnosed in 10 patients, based on clinical symptoms, namely typical precordial chest pain of class IIB or IIIB in the Braunwald classification, angiographic evidence (documented severe stenosis of 75% according to the American Heart Association classification in one or more major coronary artery), and changes in serum creatine kinase concentrations (no significant evidence of increase). SAP, which was observed in the remaining 41 patients, was diagnosed based on clinical symptoms, specifically typical precordial chest pain associated with exercise-induced cardiac ischemia and coronary angiographic findings (documented severe stenosis of 75% according to the American Heart Association classification, in one or more major coronary artery). The reference group consisted of 19 control participants (without CAD).

### Anthropometry and laboratory tests

Anthropometric variables [height and weight] were measured in the standing position and body mass index (BMI) was calculated [=weight (kg) / height (m)^2^]. Visceral fat area (VFA) and subcutaneous fat area (SFA) were measured manually on computed tomography scan at the umbilical level according to our laboratory methods [23]. Systolic and diastolic blood pressures were measured with a standard mercury sphygmomanometer on the left or right arm in the supine position after at least 10-minute rest.

Venous blood samples were collected in the morning after overnight fast for measurement of serum creatinine, lipids, glucose, and HbA1c (Japan Diabetes Society [JDS]) at next day of admission (NC and SAP groups) and about 1 week after onset of ACS (ACS group). The value of HbA1c (%) was estimated as the National Glycohemoglobin Standardization Program (NGSP) equivalent value (%), calculated by the formula HbA1c (%) = HbA1c (JDS,%) + 0.4%. For the purpose of the present study, serum samples that were obtained at baseline from each participant were stored promptly at -20°C. After thawing the samples, serum levels of Total-APN and high-molecular weight-adiponectin (HMW-APN) were measured by enzyme-linked immunosorbent assay (ELISA) kits (Human adiponectin ELISA kit, Human HMW-adiponectin ELISA kit, Otsuka Pharmaceutical Co. Tokushima, Japan) [2,16]. Serum levels of C1q-APN (units (U) /mL) and C1q (μg/mL) were measured by our in-house ELISA, as reported previously by our group [17]. The intra- and inter-coefficients of variation (CV) for C1q-APN ELISA are below 4.6% and 6.7%, respectively. The intra- and inter-CV for C1q ELISA are below 4.6% and 5.0%, respectively.

Hypertension was defined as systolic blood pressure ≥140 mmHg, and/or diastolic blood pressure ≥90 mmHg, or current treatment for hypertension. Diabetes mellitus was defined according to the World Health Organization criteria, or non-fasting plasma glucose concentration ≥126 mg/dL, and/or current treatment for diabetes mellitus. Dyslipidemia was defined as low-density lipoprotein-cholesterol concentration of ≥140 mg/dL, triglyceride concentration ≥150 mg/dL, high-density lipoprotein-cholesterol concentration <40 mg/dL, and/or treatment for dyslipidemia.

### Statistical analysis

Data are presented as mean±SEM. Differences in frequencies were examined by the χ^2^ test. Differences among groups were compared by one- or two-way analysis of variance (ANOVA) with Fisher's protected least significant difference test for multiple-group analysis. In all cases, *p* values <0.05 were considered statistically significant. All analyses were performed with the JMP Statistical Discovery Software 9.0 (SAS Institute, Cary, NC).

## Results

### Characteristics of all patients

Table 1 summarizes the characteristics of the participating subjects. The affected coronary artery was the left main coronary artery (LMCA) in 2 patients, left anterior descending artery (LAD) in 64 patients, left circumflex artery (LCX) in 20 patients and right coronary artery (RCA) in 33 patients. Single and multiple vessel disease was identified in 86% (none/single/double/triple = 19/72/32/15).

**Table 1:** Baseline characteristics of the subjects enrolled in the present study

	**All**	**NC group**	**SAP group**	**ACS group**
Number	138	19	41	78
Age, years	65 ± 1 (40–86)	65 ± 2 (51–79)	65 ± 1 (49–81)	65 ± 1 (40–86)
Body mass index, kg/m^2^	24.3 ± 0.3 (17.7-38.4)	24.3 ± 1.0 (19.5-32.5)	24.6 ± 0.6 (17.7-31.2)	24.2 ± 0.4 (17.8-38.4)
Visceral fat area, cm^2^	117 ± 5 (25–223)	101 ± 13 (25–223)	125 ± 10 (17–277)	116 ± 6 (18–259)
Subcutaneous fat area, cm^2^	130 ± 5 (22–361)	124 ± 15 (45–329)	142 ± 8 (50–224)	126 ± 7 (22–361)
Smoking (none-/ex-/current-smoker), n	45/32/61	9/5/5	14/10/17*	22/17/39
Diabetes mellitus, n	69 (50%)	10 (53%)	29 (71%)	30 (38%)
sulfonyl ureas/glinides/biguanides/alpha glucosidase inhibitors/Insulin, n	22/5/10/20/6	5/0/1/2/2	10/5/8/2/2	7/0/1/16/2
Hypertension, n	92 (67%)	15 (79%)	27* (66%)	50 (64%)
calcium channel antagonists/angiotensin receptor blockers /β-blockers/diuretics, n	44/70/62/9	7/2/2/0	21/20/4/4	16/48/56/5
Dyslipidemia, n	61 (44%)	7 (37%)	24* (59%)	30 (38%)
Statins/fibrates/ezetimibe/cholestimide, n	83/2/1/1	10/1/0/0	10/0/1/1	63/1/0/0
anti-platelet drugs (aspirin/ticlopidine/clopidogrel), n	115/32/65	6/0/4	31/0/20	78/32/41
Family history of CAD, n	13 (9%)	2 (11%)	3* (7%)	8 (10%)
Systolic blood pressure, mmHg	138 ± 2 (90–217)	139 ± 4 (108–173)	138 ± 3 (100–175)	138 ± 3 (90–217)
Diastolic blood pressure, mmHg	83 ± 1 (49–146)	79 ± 2 (64–94)	75 ± 2 (49–99)	87 ± 2 (57–146)^†^
Hemoglobin A1c (NGSP), %	6.7 ± 0.1 (3.4-12.2)	7.3 ± 0.4 (5.8-9.8)	6.9 ± 0.2 (5.5-10.1)	6.6 ± 0.2 (3.4-12.2)
LDL-C, mg/dL	112 ± 3 (20–250)	114 ± 8 (58–154)	107 ± 6 (49–197)	115 ± 4 (20–250)
Triglyceride, mg/dL	122 ± 7 (24–611)	124 ± 16 (49–263)	140 ± 12 (59–374)	113 ± 10 (24–611)
HDL-C, mg/dL	49 ± 1 (26–128)	63 ± 4 (39–95)	53 ± 3 (27–104)	44 ± 2 (26–128)^¶,†^
Creatinine, mg/dL	0.85 ± 0.03 (0.47-1.34)	0.86 ± 0.04 (0.60-1.20)	0.85 ± 0.03 (0.60-1.30)	0.91 ± 0.04 (0.47-1.34)
Target lesions (LMCA/LAD/LCX/RCA), n	2/64/20/33	-	2/20/14/5	0/44/6/28
Vessels (None/SVD/DVD/TVD), n	19/72/32/15	-	0/22/14/5	0/50/18/10
Procedures (PCI/CABG), n	118/1	-	40/1	78/0

Data are mean ± SEM (range), or number of subjects analyzed. *P < 0.05, Compared with the NC group, ^¶^P < 0.0001, Compared with the NC group, ^†^P < 0.001, Compared with the SAP group.

Differences among groups were compared by one- or two-way analysis of variance (ANOVA) with Fisher's protected least significant difference test for multiple-group analysis. Differences in frequencies were examined by the χ^2^ test. CAD, Coronary artery disease; HDL-C, High-density lipoprotein-cholesterol; LDL-C, Low-density lipoprotein-cholesterol; LMCA, Left main coronary artery; LAD, Left anterior descending artery; LCX, Left circumflex artery; RCA, Right coronary artery; SVD, Single vessel disease; DVD, Double vessels disease; TVD, Triple vessels disease; PCI, Percutaneous coronary intervention; CABG, Coronary artery bypass graft.

### Comparison of adiponectin parameters among NC, SAP and ACS groups

**Figure 1:**
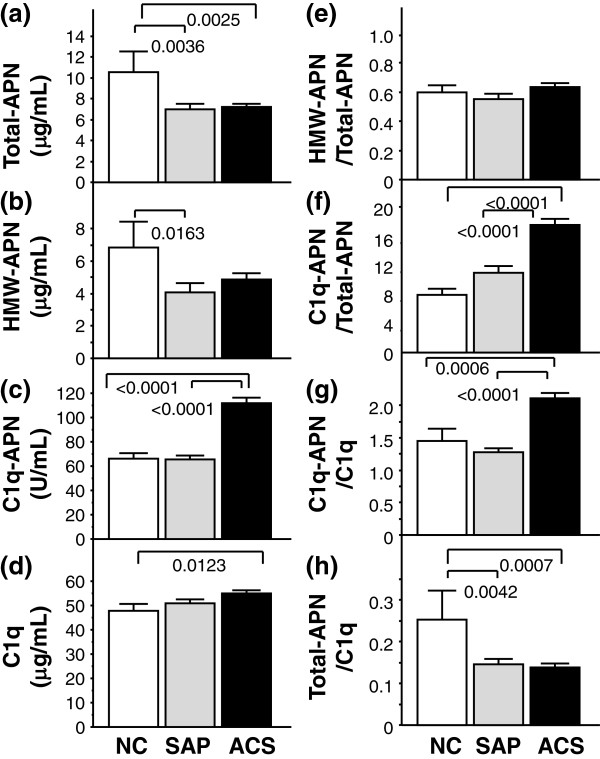
**Circulating levels of (a) Total-APN, (b) HMW-APN, (c) C1q-APN, (d) C1q, (e) HMW-APN/Total-APN, (f) C1q-APN/Total-APN, (g) C1q-APN/C1q, and (h) Total-APN/C1q in subjects without CAD (Normal coronary; NC), stable angina pectoris (SAP) and acute coronary syndrome (ACS).** Differences among groups were compared by one- or two-way analysis of variance (ANOVA) with Fisher's protected least significant difference test for multiple-group analysis.

Table 1 shows the characteristics of patients without CAD, with SAP and those with ACS. There were no significant differences in age, BMI, VFA and SFA between the three groups, which is known to influence serum levels of Total-APN [2] and C1q-APN [17]. Subjects with SAP had higher prevalence of current smoke, hypertension, dyslipidemia, than those with NC. Serum HDL-C levels were significantly lower in the ACS group, compared with the NC and SAP groups. Serum Total-APN levels were significantly lower in the SAP (7.0±0.5 μg/mL) and ACS (7.2±0.3) groups, compared with the NC group (10.6±2.0, Figure 1a). Serum HMW-APN levels were significantly lower in the SAP group (4.1±0.5 μg/mL) and trended to be lower in ACS group (4.9±0.4), compared with the NC group (6.8±1.6, Figure 1b). Serum C1q-APN levels were significantly higher in ACS group (112.1±4.1 U/mL) compared with the NC (66.3±4.4) and SAP group (65.7±2.9, Figure 1c). Serum C1q levels were significantly higher in the ACS group (54.9±1.2 μg/mL), compared with the NC group (48.0±2.5, Figure 1d). There was no significant difference in serum HMW-APN/Total-APN ratio among the three groups (ACS: 0.60±0.04, SAP: 0.55±0.04, NC: 0.63±0.03, Figure 1e). Serum C1q-APN/Total-APN ratio was significantly higher in the ACS group (17.50±0.83), compared with the NC (7.96±0.81) and SAP (10.95±0.89, Figure 1f) groups. Serum C1q-APN/Total-APN ratio was significantly higher in the SAP group than in the NC group (Figure 1f) Serum C1q-APN/C1q ratio was significantly higher in the ACS group (2.11±0.09), compared with the NC (1.28±0.05) and SAP (1.45±0.16, Figure 1g) groups. Serum Total-APN/C1q ratio was significantly lower in the SAP (0.15±0.01) and ACS (0.14±0.01) groups, compared with the NC group (0.25±0.07, Figure 1h).

## Discussion

The following were the major findings of the present study: 1) serum C1q-APN levels were higher in the ACS group than in the NC and SAP groups, 2) serum C1q levels were higher in the ACS group than in the NC group, and 3) serum C1q-APN/Total-APN ratio was higher in the SAP and ACS groups, compared with the NC group.

This is the first report of significantly higher serum C1q levels in patients with ACS compared with the control (Figure 1d). The complement system is part of the immune system in acute phase ACS [1]. C1q interacts with complement cell-surface receptor to promote phagocytosis and local pro-inflammatory response [24]. Pathogen associated molecular pattern (PAMP) recognized and bound by defense collagens, such as C1q, leads to rapid containment of infection via complement activation [25]. The inflammatory response is also mediated by the patient's innate immune cells including adipocyte apoptosis, which are equipped with pattern recognition receptors. These receptors are able to sense injury-induced, damage-associated molecular patterns (DAMPs) generated during collection, processing, and storage of blood/blood components [26]. These systems may promote the formation of acute and chronic phase of atherosclerosis.

On the other hand, adiponectin shows structural homology to collagen VIII, X and complement factor C1q [3] and belongs to soluble defense collagen family [27]. Peake et al. reported that adiponectin bound C1q in vitro, which induces activation of the classical complement pathway [28]. Adiponectin have been shown to facilitate enhanced phagocytosis and modulate induction of cytokines towards an anti-inflammatory profile and protect against cardiovascular diseases [29]. Adiponectin increases the expression of tissue inhibitor of metalloproteinase-1 in macrophages [30], thus may inhibit matrix degradation in atheromatous plaques. In the present study, hypoadiponectinemia was detected in patients with ACS and SAP (low serum Total-APN levels) (Figure 1a), in agreement with several previous studies [10-15]. We recently reported that serum C1q-APN/Total-APN ratio correlated with the metabolic syndrome in men [17], and with polyvascular diseases and CAD in type 2 diabetics [18,19], although there was no significant difference in serum C1q-APN levels between patients without and with CAD. The present study also showed no significant difference in serum C1q-APN levels between NC and SAP groups. Interestingly, the present study demonstrated that serum C1q-APN levels were 1.7-fold higher in patients with ACS than those of the NC and SAP groups (Figure 1c). The ACS groups had significantly lower serum HDL-C levels, than the NC and SAP groups (Table 1). Our previous report had reported that stepwise multiple regression analysis did not identify HDL-C as a significant determinant of C1q–APN [17]. Some studies reported that serum complement levels were markedly elevated in ACS compared with SAP patients, suggesting an acute phase and inflammatory response [31,32]. The protein family CTRPs (C1q/TNF-related proteins) has recently been identified as adiponectin paralogs and some CTRP members share adiponectin's metabolic regulatory function and regulate cardiac remodeling after acute myocardial infarction [33,34]. These results suggest that adiponectin seems to have a protective role in activated complement system in the development of ACS and have an impact on the self-defense system through its binding with C1q in blood. Measurements of serum Total-APN plus C1q-APN may become instrumental to combat ACS. However, to date, the precise value of serum C1q-APN cannot be measured, because the proportion of blood adiponectin that forms protein complex with C1q remains unclear. Further clinical and experimental studies are needed to clarify the role of adiponectin and C1q network in ACS.

In conclusion, the present study indicates that serum C1q-APN levels were higher in male patients with ACS, and that serum C1q-APN/Total-APN ratio was higher in male patients with SAP and ACS than the control. Serum C1q-APN might be a promising target in attempts to reduce ACS.

### Study limitations

The present study has several limitations. First, this is a cross-sectional study, making it difficult to establish a cause-effect relationship. Further prospective studies should be conducted to analyze this relationship. Second, all patients in this study were Japanese men and any differences from other ethnic groups are unknown. All participants were Japanese and each gave a written informed consent. Based upon 80% power to detect statistically significant differences (p = 0.05; two-sided) as our group reported previously [18,19], a sample size of at least 20 patients in each group was required to demonstrate (total sample size = 60). Further multicenter studies that include larger samples of subjects, especially those of other races, are needed. Finally, we measured various laboratory parameters and fat distribution at about one week after onset of ACS, therefore, medical therapy for ACS may have affected these parameters [35].

## Abbreviations

ACS: Acute coronary syndrome; AMI: Acute myocardial infarction; BMI: Body mass index; CAD: Coronary artery disease; C1q-APN: C1q-binding adiponectin; ELISA: Enzyme-linked immunosorbent assay; HMW-APN: High-molecular weight adiponectin; NC: Normal coronary; SAP: Stable angina pectoris; SFA: Subcutaneous fat area; TIMI: Thrombolysis in myocardial infarction; Total-APN: Total-adiponectin; UAP: Unstable angina pectoris; VFA: Visceral fat area.

## Competing interests

TF is a member of the “Department of Metabolism and Atherosclerosis”, a sponsored course endowed by Kowa Co. Ltd.. The company has a scientific officer who oversees the program. All other authors declare no competing interests. Human serum C1q-binding adiponectin complex assay is under patent application in Japan.

## Authors’ contributions

KK researched and analyzed the data, participated in the concept and design of the study, interpretation of data and reviewed/edited the manuscript. HK analyzed the data. YN, TM, HY and KY recruited the patients and collected the data. TF and IS contributed to the discussion. All authors read and approved the final version of the manuscript.

## References

[B1] Correale M Brunetti ND De Gennaro L Di Biase M Acute phase proteins in atherosclerosis (acute coronary syndrome) Cardiovasc Hematol Agents Med Chem 2008 6 272 277 10.2174/187152508785909537 18855639

[B2] Arita Y Kihara S Ouchi N Takahashi M Maeda K Miyagawa J Hotta K Shimomura I Nakamura T Miyaoka K Kuriyama H Nishida M Yamashita S Okubo K Matsubara K Muraguchi M Ohmoto Y Funahashi T Matsuzawa Y Paradoxical decrease of an adipose-specific protein, adiponectin, in obesity Biochem Biophys Res Commun 1999 257 79 83 10.1006/bbrc.1999.0255 10092513

[B3] Okamoto Y Arita Y Nishida M Muraguchi M Ouchi N Takahashi M Igura T Inui Y Kihara S Nakamura T Yamashita S Miyagawa J Funahashi T Matsuzawa Y An adipocyte-derived plasma protein, adiponectin, adheres to injured vascular walls Horm Metab Res 2000 32 47 50 10.1055/s-2007-978586 10741683

[B4] Matsuda M Shimomura I Sata M Arita Y Nishida M Maeda N Kumada M Okamoto Y Nagaretani H Nishizawa H Kishida K Komuro R Ouchi N Kihara S Nagai R Funahashi T Matsuzawa Y Role of adiponectin in preventing vascular stenosis. The missing link of adipo-vascular axis J Biol Chem 2002 277 37487 37491 10.1074/jbc.M206083200 12138120

[B5] Okamoto Y Kihara S Ouchi N Nishida M Arita Y Kumada M Ohashi K Sakai N Shimomura I Kobayashi H Terasaka N Inaba T Funahashi T Matsuzawa Y Adiponectin reduces atherosclerosis in apolipoprotein E-deficient mice Circulation 2002 106 2767 2770 10.1161/01.CIR.0000042707.50032.19 12451000

[B6] Kishida K Funahashi T Shimomura I Molecular mechanisms of diabetes and atherosclerosis: role of adiponectin Endocr Metab Immune Disord Drug Targets 2012 12 118 131 10.2174/187153012800493468 22236026

[B7] Baldasseroni S Antenore A Di Serio C Orso F Lonetto G Bartoli N Foschini A Marella A Pratesi A Scarantino S Fumagalli S Monami M Mannucci E Marchionni N Tarantini F Adiponectin, diabetes and ischemic heart failure: a challenging relationship Cardiovasc Diabetol 2012 11 151 10.1186/1475-2840-11-151 23249664 PMC3558365

[B8] Ye R Scherer PE Adiponectin, driver or passenger on the road to insulin sensitivity? Mol Metab 2013 2 133 141 10.1016/j.molmet.2013.04.001 24049728 PMC3773837

[B9] Hotta K Funahashi T Arita Y Takahashi M Matsuda M Okamoto Y Iwahashi H Kuriyama H Ouchi N Maeda K Nishida M Kihara S Sakai N Nakajima T Hasegawa K Muraguchi M Ohmoto Y Nakamura T Yamashita S Hanafusa T Matsuzawa Y Plasma concentrations of a novel, adipose-specific protein, adiponectin, in type 2 diabetic patients Arterioscler Thromb Vasc Biol 2000 20 1595 1599 10.1161/01.ATV.20.6.1595 10845877

[B10] Ouchi N Kihara S Arita Y Maeda K Kuriyama H Okamoto Y Hotta K Nishida M Takahashi M Nakamura T Yamashita S Funahashi T Matsuzawa Y Novel modulator for endothelial adhesion molecules: adipocyte-derived plasma protein adiponectin Circulation 1999 100 2473 2476 10.1161/01.CIR.100.25.2473 10604883

[B11] Kumada M Kihara S Sumitsuji S Kawamoto T Matsumoto S Ouchi N Arita Y Okamoto Y Shimomura I Hiraoka H Nakamura T Funahashi T Matsuzawa Y Osaka CAD Study Group Coronary artery disease. Association of hypoadiponectinemia with coronary artery disease in men Arterioscler Thromb Vasc Biol 2003 23 85 89 10.1161/01.ATV.0000048856.22331.50 12524229

[B12] Nakamura Y Shimada K Fukuda D Shimada Y Ehara S Hirose M Kataoka T Kamimori K Shimodozono S Kobayashi Y Yoshiyama M Takeuchi K Yoshikawa J Implications of plasma concentrations of adiponectin in patients with coronary artery disease Heart 2004 90 528 533 10.1136/hrt.2003.011114 15084551 PMC1768199

[B13] Otsuka F Sugiyama S Kojima S Maruyoshi H Funahashi T Matsui K Sakamoto T Yoshimura M Kimura K Umemura S Ogawa H Plasma adiponectin levels are associated with coronary lesion complexity in men with coronary artery disease J Am Coll Cardiol 2006 48 1155 1162 10.1016/j.jacc.2006.05.054 16978998

[B14] Liang KW Sheu WH Lee WL Liu TJ Ting CT Hsieh YC Wang KY Chen YT Lee WJ Decreased circulating protective adiponectin level is associated with angiographic coronary disease progression in patients with angina pectoris Int J Cardiol 2008 129 76 80 10.1016/j.ijcard.2007.05.027 17651832

[B15] Schnabel R Messow CM Lubos E Espinola-Klein C Rupprecht HJ Bickel C Sinning C Tzikas S Keller T Genth-Zotz S Lackner KJ Münzel TF Blankenberg S Association of adiponectin with adverse outcome in coronary artery disease patients: results from the AtheroGene study Eur Heart J 2008 29 649 657 10.1093/eurheartj/ehn009 18263867

[B16] Komura N Kihara S Sonoda M Kumada M Fujita K Hiuge A Okada T Nakagawa Y Tamba S Kuroda Y Hayashi N Sumitsuji S Kawamoto T Matsumoto S Ouchi N Arita Y Okamoto Y Shimomura I Funahashi T Matsuzawa Y Osaka CAD Group Clinical significance of high-molecular weight form of adiponectin in male patients with coronary artery disease Circ J 2008 72 23 28 10.1253/circj.72.23 18159094

[B17] Nakatsuji H Kobayashi H Kishida K Nakagawa T Takahashi S Tanaka H Akamatsu S Funahashi T Shimomura I Binding of adiponectin and C1q in human serum, and clinical significance of the measurement of C1q-adiponectin/total adiponectin ratio Metabolism 2013 62 109 120 10.1016/j.metabol.2012.06.006 22819529

[B18] Hirata A Kishida K Kobayashi H Nakatsuji H Funahashi T Shimomura I Correlation between serum C1q-adiponectin/total adiponectin ratio and polyvascular lesions detected by vascular ultrasonography in Japanese type 2 diabetics Metabolism 2013 62 376 385 10.1016/j.metabol.2012.08.009 23058931

[B19] Hirata A Kishida K Nakatsuji H Kobayashi H Funahashi T Shimomura I High serum C1q-adiponectin/total adiponectin ratio correlates with coronary artery disease in Japanese type 2 diabetics Metabolism 2013 62 578 585 10.1016/j.metabol.2012.10.011 23174407

[B20] Nakatsuji H Kishida K Funahashi T Shimomura I Senri Study II Group Three-month treatment with pioglitazone reduces circulating levels of thiobarbituric acid-reacting substances, a marker of reactive oxidative stress, without change in body mass index, in Japanese patients with type 2 diabetes Atherosclerosis 2010 212 243 245 10.1016/j.atherosclerosis.2010.05.025 20541758

[B21] Nakatsuji H Kishida K Kobayashi H Funahashi T Shimomura I Senri Study II Group Three-month treatment with pioglitazone reduces circulating C1q-binding adiponectin complex to total-adiponectin ratio, without changes in body mass index, in type 2 diabetics Diabetes Res Clin Pract 2013 99 e14 17 10.1016/j.diabres.2012.10.003 23142017

[B22] Kishida K Kishida N Arima M Nakatsuji H Kobayashi H Funahashi T Shimomura I Serum C1q- binding adiponectin in maintenance hemodialysis patients BMC Nephrol 2013 14 50 10.1186/1471-2369-14-50 23442371 PMC3598349

[B23] Yoshizumi T Nakamura T Yamane M Islam AH Menju M Yamasaki K Arai T Kotani K Funahashi T Yamashita S Matsuzawa Y Abdominal fat: standardized technique for measurement at CT Radiology 1999 211 283 286 10.1148/radiology.211.1.r99ap15283 10189485

[B24] Kishore U Reid KB C1q: structure, function, and receptors Immunopharmacology 2000 49 159 170 10.1016/S0162-3109(00)80301-X 10904115

[B25] Fraser DA Tenner AJ Directing an appropriate immune response: the role of defense collagens and other soluble pattern recognition molecules Curr Drug Targets 2008 9 113 122 10.2174/138945008783502476 18288962

[B26] Tang D Kang R Coyne CB Zeh HJ Lotze MT PAMPs and DAMPs: signal 0 s that spur autophagy and immunity Immunol Rev 2012 249 158 175 10.1111/j.1600-065X.2012.01146.x 22889221 PMC3662247

[B27] Yokota T Oritani K Takahashi I Ishikawa J Matsuyama A Ouchi N Kihara S Funahashi T Tenner AJ Tomiyama Y Matsuzawa Y Adiponectin, a new member of the family of soluble defense collagens, negatively regulates the growth of myelomonocytic progenitors and the functions of macrophages Blood 2000 96 1723 1732 10961870

[B28] Peake PW Shen Y Walther A Charlesworth JA Adiponectin binds C1q and activates the classical pathway of complement Biochem Biophys Res Commun 2008 367 560 565 10.1016/j.bbrc.2007.12.161 18179772

[B29] Takemura Y Walsh K Ouchi N Adiponectin and cardiovascular inflammatory responses Curr Atheroscler Rep 2007 9 238 243 10.1007/s11883-007-0025-4 18241619

[B30] Kumada M Kihara S Ouchi N Kobayashi H Okamoto Y Ohashi K Maeda K Nagaretani H Kishida K Maeda N Nagasawa A Funahashi T Matsuzawa Y Adiponectin specifically increased tissue inhibitor of metalloproteinase-1 through interleukin-10 expression in human macrophages Circulation 2004 109 2046 2049 10.1161/01.CIR.0000127953.98131.ED 15096450

[B31] Giasuddin AS ElMahdawi JM ElHassadi FM Serum complement (C3, C4) levels in patients with acute myocardial infarction and angina pectoris Bangladesh Med Res Counc Bull 2007 33 98 102 18783065 10.3329/bmrcb.v33i3.1141

[B32] Iltumur K Karabulut A Toprak G Toprak N Complement activation in acute coronary syndromes APMIS 2005 113 167 174 10.1111/j.1600-0463.2005.apm1130303.x 15799759

[B33] Zheng Q Yuan Y Yi W Lau WB Wang Y Wang X Sun Y Lopez BL Christopher TA Peterson JM Wong GW Yu S Yi D Ma XL C1q/TNF-related proteins, a family of novel adipokines, induce vascular relaxation through the adiponectin receptor-1/AMPK/eNOS/nitric oxide signaling pathway Arterioscler Thromb Vasc Biol 2011 31 2616 2623 10.1161/ATVBAHA.111.231050 21836066 PMC3197867

[B34] Sun Y Yi W Yuan Y Lau WB Yi D Wang X Wang Y Su H Wang X Gao E Koch WJ Ma XL C1q/tumor necrosis factor-related protein-9, a novel adipocyte-derived cytokine, attenuates adverse remodeling in the ischemic mouse heart via protein kinase A activation Circulation 2013 128 S113 120 10.1161/CIRCULATIONAHA.112.000010 24030394 PMC3824619

[B35] Kojima S Funahashi T Sakamoto T Miyamoto S Soejima H Hokamaki J Kajiwara I Sugiyama S Yoshimura M Fujimoto K Miyao Y Suefuji H Kitagawa A Ouchi N Kihara S Matsuzawa Y Ogawa H The variation of plasma concentrations of a novel, adipocyte derived protein, adiponectin, in patients with acute myocardial infarction Heart 2003 89 667 10.1136/heart.89.6.667 12748233 PMC1767676

